# Comment on Cobo-Cuenca, A.I.; Laredo-Aguilera, J.A.; Rodríguez-Borrego, M.-A.; Santacruz-Salas, E.; Carmona-Torres, J.M. Temporal Trends in Fecal Occult Blood Test: Associated Factors (2009–2017). *Int. J. Environ. Res. Public Health* 2019, *16*, 2120

**DOI:** 10.3390/ijerph16245008

**Published:** 2019-12-09

**Authors:** Isabel Portillo

**Affiliations:** Osakidetza Basque Health Service & Biocruces Bizkaia Health Research Institute, Bilbao, Bizkaia, 48011 Bilbao, Spain; mariaisabel.portillovillares@osakidetza.eus

I have carried out an in depth reading of the article by Cobo-Cuenca et al 2019 [1]. entitled “Temporal trends in fecal occult blood test: associated factors (2009–2017)”. The authors provide information on the evolution of colorectal cancer screening participation using data from the European Health Survey and National Health Survey in Spain. 

Among the results they include disaggregated data on the situation in each of the 17 autonomous communities (see Table 3 in their article). It seems confusing to provide percentages by row, as what would really inform on the evolution in participation in fecal occult blood test (FOBT) would be the percentages of FOBT participants among all respondents from 50 to 70 years old each year. Additionally, as different surveys have been examined together, I am in doubt whether results are weighted, which entails a questionable representativeness in the smaller autonomous communities.

Moreover, the aforementioned table seems to include FOBT for any reason, although the authors discuss the results as if they were all part of screening programs. In the case of País Vasco, according to the regional health survey conducted in 2018, there was an 89.0% self-reported participation among invitees to take part in the colorectal cancer screening programme with FOBT [2].

If the focus is on participation in colorectal cancer screening programs only, as the authors point out, screening implementation is unequal across Spain. There is a wide range of participation, going from 22.7% in Andalucía to 72.4% in País Vasco in 2016 [3]. However, this should be contextualized as the program coverage is unequal too. It is complete in País Vasco and Navarra, while in Canarias it is expected to be so in 2024 [4].

Additionally, the surveys include information on doing a colonoscopy, which is a factor that can involve the recommendation of not being screened with a FOBT. Moreover, confidence intervals are not included in the paper, which is a bias in the analysis.

Thus, I consider this article must be carefully reviewed by your editorial board, reviewers and authors in order to correct the inappropriate methods, inaccurate results and conclusions.
